# Interdisciplinary Pathways to Death Literacy: Social Model Hospice Homes as a Site for Experiential Learning

**DOI:** 10.1093/geroni/igaf122.2097

**Published:** 2025-12-31

**Authors:** Kelly Melekis, Carol Weisse

**Affiliations:** University of Vermont, Burlington, Vermont, United States; Union College, Schenectady, New York, United States

## Abstract

Over the last century, major shifts have occurred in the way people die, including where they die and who provides most of the care. Most people wish to die at home, and while home deaths are more likely to result in patient-centered, goal-concordant care (Khandelwal et al., 2017), only 30% of people in the U.S. die at home (Cross & Warraich, 2019). Several barriers prevent individuals from dying in their preferred place, including caregiver and/or housing instability as well as a lack of knowledge and skills needed to make informed decisions. In this presentation, social model hospice homes will be discussed as a resource for addressing gaps in end-of-life care and improving death literacy. Death literacy, or knowledge and understanding about death and end-of-life care, impacts access to quality care for dying people (Collins et al., 2020) and allows a community approach to supporting those experiencing death, dying, and bereavement. As a community-driven response to end-of-life care needs, social model hospice provides access to hospice services in a home setting with support of volunteer community caregivers (Kliewer et al., 2023). This presentation will share lessons learned from a 10-year, multi-site collaboration to enhance death literacy through 1) research practice partnerships between community-run social model hospice homes, undergraduate student caregivers, and thanatology researchers, and 2) an experiential learning Community Action, Research, and Education (CARE) program whereby participants care for hospice patients in their last months of life. Strategies for developing interdisciplinary research-practice partnerships and experiential learning opportunities will be discussed.

